# Podcast Use in Undergraduate Medical Education

**DOI:** 10.7759/cureus.1930

**Published:** 2017-12-09

**Authors:** Alvin Chin, Anton Helman, Teresa M Chan

**Affiliations:** 1 Department of Medicine, Division of Emergency Medicine, McMaster University; 2 Department of Family and Community Medicine, University of Toronto; 3 Faculty of Health Sciences, Department of Medicine, Division of Emergency Medicine, McMaster University

**Keywords:** podcast, medical education, foam

## Abstract

Introduction

Podcasts have become increasingly popular as a medium for free open access medical education (FOAM). However, little research has examined the use of these extracurricular audio podcasts as tools in undergraduate medical education. We aimed to examine knowledge retention, usage conditions, and preferences of undergraduate medical students at a Canadian university interacting with extracurricular podcasts.

Methods

Students enrolled in the undergraduate medical program at McMaster University volunteered to participate in this study. Two podcasts were created specifically for the purposes of this study, and online tests and surveys were sent to participants to gather data regarding user preferences of podcasts. In addition, we recorded changes in topic test scores before and after podcast exposure.

Results

Forty-two students were recruited to this study. Participants who completed the assessments demonstrated an effect of learning. Podcasts of 30 minutes or less were preferred in the majority of participants who had a preference in duration. The top three activities participants were engaged in while listening to the podcasts were driving (46%), completing chores (26%), and exercising (23%). A large number of participants who did not complete the study in its entirety cited a lack of time and podcast length to be the top two barriers to completion.

Conclusion

This is one of the first studies to examine extracurricular podcast-usage data and preferences in a Canadian undergraduate medical student population. This information may help educators and FOAM producers to optimize educational tools for medical education.

## Introduction

Podcasts are one of many modalities that have become increasingly popular as an alternative medium for learning associated with the free open access medical education (FOAM) movement [1]. Previous studies have shown that podcasts may be preferred by trainees more than textbooks for learning [2-3]. When asked, medical students state that having podcasts as an alternative resource or adjunct for their learning is beneficial [2, 4]. Perceived advantages to having access to podcasts include: reducing stress and anxiety, ability to engage in other activities while learning, and the ability to cater to students with varying study habits [5-6]. Furthermore, one recent study even suggests that when compared to reading a book chapter, podcasts may result in increased knowledge retention [7].

Much of the current research mainly investigates podcasts that incorporate a visual component but few have studied audio-only podcasts [2, 4-5, 7]. Numerous studies have examined the acceptability or feasibility of this modality for learning by various groups (predominantly in resident physician populations), however, limited studies have focused on the use of these tools for extracurricular learning, particularly in the undergraduate medical education setting [8-10].

With the increasing abundance of FOAM resources, learners are often using podcasts for learning in an extracurricular fashion – i.e., they are reading and learning on top of their curricular needs. A recent survey of emergency medicine (EM) residents showed that they used podcasts to keep up to date with the literature and to learn core content [8]. Similarly, podcasts and other FOAM have been reported by residents as being used in addition to their regular curriculum through training [3, 8]. Little research has examined the use of audio podcasts as a tool for extracurricular learning in undergraduate medical education. By understanding audio podcast usage conditions and preferences of medical students, FOAM educators may be able to better target this audience.

We aimed to determine usage conditions, preferences, and level of retention of information from audio podcasts by medical students at a Canadian University.

## Materials and methods

Study design

This was a prospective study aimed towards assessing knowledge retention, usage conditions, and preferences of students interacting with two pre-determined podcasts. This study was approved by the Hamilton Integrated Research Ethics Board.

Setting and selection of participants

The study recruited participants out of McMaster University medical school’s three undergraduate classes. Each undergraduate class had approximately 200 students, for a total of approximately 600 students eligible to participate. Recruitment occurred between September 14, 2015 to September 28, 2015. All three classes were notified about this study through the undergraduate medical school program’s master email list. Students actively enrolled in the McMaster undergraduate medical program were allowed to participate without any exclusion. Participants voluntarily signed up for this study and there was no incentive given to complete the study. Participation in this study did not affect the academic standing of students.

Protocol

Two audio podcasts were created collaboratively by the three authors of this paper. Two of the authors were part of a popular FOAM podcast series (Emergency Medicine Cases, https://emergencymedicinecases.com/). The podcasts covered two topics: adult asthma and introduction to toxicology. The topic of toxicology was specifically selected as it is not well covered in the official curriculum at this educational institution, thereby allowing this podcast to provide the students with more primary education. The length of the asthma and toxicology podcasts were 58 minutes and 37 minutes, respectively.

Along with the podcasts, a test was created for each topic, collaboratively, by the three authors. Each test consisted of 10-11 multiple choice questions (MCQ) all stemming from material covered in the podcast, relevant to the individual topics. The questions were not externally validated but were reviewed by two emergency staff physicians.

Following enrolment, participants of this study were instructed to complete the two online tests, mentioned above, and a survey in order to assess their baseline knowledge on the topics of the podcasts, and podcast usage and preferences. The two podcasts were then distributed to study participants following the initial test and survey.

One week after the initial survey, students were asked to complete a follow-up survey and test for knowledge assessment and further podcast usage data. This follow-up test used the exact same questions as the initial test. Students were only eligible to write the test if they had indicated that they had listened to the podcast associated with the test. Participants were reminded within 48 hours after receiving the link to complete the survey/test via email.

Survey questions included both categorical and open-ended questions. Students were asked questions regarding the number of times they interacted with the podcasts, the activities they were engaged in while listening, and duration preferences. Open-ended questions such as “What did you like about the podcast?” and “What would have helped you most with consolidating the material?” were included to allow for broader responses. In addition, students were allowed to state barriers to interacting with the podcast regardless of whether or not they listened to it.

Primary data analysis

Paired samples t-tests were utilized to assess knowledge acquisition and retention using Microsoft Statistical Package for the Social Sciences (SPSS), version 23 (IBM Corp., Armonk, NY). Simple descriptive statistics related to the survey responses were generated using Microsoft Excel. We marked each round of the MCQ quiz and converted this to a percentage score for each round.

## Results

Participation

A total of 42 (11 first year, 17 second year, and 14 third year) students volunteered to participate in this study and completed the initial survey and baseline knowledge test. They were then also provided with links to two audio files (one with an audio file with the Asthma Podcast, and one with the Toxicology Podcast). Only 19 (45.2%) students completed a follow-up knowledge retention test for the asthma podcast and 14 (33.3%) for the toxicology podcast.

Thirty-five students spoke to the barriers of listening to the podcasts regardless of whether or not they listened to either. The most cited reason for incompletion of the survey/test was a lack of time and podcast length (81%). Other reasons included technical problems and formal curriculum commitments.

Due to the nature of access to the podcast being a downloadable format, we were unable to determine the number of times each podcast was listened to by each user.

Survey data

Quantitative Survey Responses

Thirteen participants indicated that they had a preference for podcast length. The majority of participants who stated a preference in podcast length indicated they preferred podcasts of 30 minutes or less (85%).

The top three activities participants were engaged in while listening to the podcasts were driving (46%), completing chores (26%), and exercising (23%). Other activities listed included eating, doing other work, using other computer applications, and other recreational activities. Twenty-percent of respondents stated that they had at some point solely focused on podcast engagement. Note that the percentage response adds to over 100% as participants interacted with podcasts more than once. See Table 1 for details of tasks that were competing for the students’ attention during this period.

**Table 1:** Participant activities Activities participants were engaged in while listening to podcasts.

Activity	n	%	Example responses
Driving	16	46%	n/a
Chores	9	26%	Cooking, Dishes, Ironing, Getting Ready in the Morning
Exercising	8	23%	Walk or high intensity
Only Engaging with Podcast (Listening, Taking notes)	7	20%	n/a
Eating	6	17%	n/a
Doing Other Work	4	11%	Other curriculum based homework
Using Other Computer Apps	2	6%	Social Media
Other recreation	1	3%	Drawing
* Will total over 100% since many people reported doing multiple activities over course of listening session			

Qualitative Survey Responses

In the open-ended questions, students noted that they liked that the podcasts were “conversational” and appreciated “the clinical relevance of the information” and use of cases. A common point of discontent was that the podcasts were “too long”. Finally, the majority of students indicated that a “summary at the end” or “visuals to go along with [the] discussion” would help benefit them in retaining the information.

Knowledge Acquisition

Participants who successfully completed the knowledge assessments demonstrated a significant effect of learning (Asthma, baseline score: 60%, post-podcast score: 76% (95% CI: 68-83); Toxicology, baseline score: 62%, post-podcast score: 82% (95% CI: 72-92)). See Table 2 for further details of the analysis.

**Table 2:** Pre and post-test analysis Paired t-test analysis of pre and post-test. Both groups showed significant increase in post-test scores.

	Pre-test mean	Post-test mean	t-score	SD	p-value
Toxicology	62%	82%	4	0.2	0.003
Asthma	60%	76%	3.9	0.16	0.001

## Discussion

A large number recent studies in this field mainly examine the utility of ‘rich-media’ podcasts (also known as video podcasts or ‘vodcasts’), which generally include both a video and audio component to the podcast [2, 4-7, 11]. In our study, we aimed to evaluate the user preferences and habits of audio-only podcasts, commonly found in the FOAM world. Numerous studies have already demonstrated the knowledge acquisition from and desire of podcast utilization in undergraduate and postgraduate medical education [6, 8-10]. It is, therefore, essential for educators to optimize resources for medical learners in order to develop a more robust educational experience. Our findings summarize the usage of audio-only podcasts for co- or extra-curricular education in an undergraduate medical school and will hopefully guide educators in developing improved resources based on user preferences.

The realities of extracurricular learning

A total of 26 participants stated that they had not listened to at least one podcast out of the two by the end of the two-week study. Again, the recurring reason that respondents stated for being unable to do so was a lack of time (whether that was due to other life or school commitments) or that the podcasts were too long in duration. These responses, along with the participant responses to open-ended questions indicated preferences similar to other studies. In a study by Matava and colleagues, anesthesia residents similarly preferred shorter podcasts less than 30 minutes and were less likely to listen to podcasts of >45 minutes in length [10]. This preference for podcasts less than 30 minutes in length was also noted in the study by Riddell and colleagues in EM residents [8]. As a point of improvement, a number of participants noted that visual adjuncts may benefit their learning, similar to findings of Shantikumar and colleagues [6].

Potentially, a substantive effect on learning

In our study, we demonstrated that students who completed our study had a significant and likely educationally-relevant improvement in knowledge after engaging with the extracurricular audio podcasts on both the topics of asthma and toxicology. Admittedly, in our design, we did not have a pure control group, and therefore this knowledge improvement may also have been from curriculum material. However, in our design, we intentionally chose toxicology as one of the podcast topics as there is very little curricular-based education on toxicology in the formal medical school curriculum; and, hence, this effectively acted as a control, when compared to the Asthma group (where there is substantial overlap with formal curricular materials). Participants who listened to the toxicology podcast and completed a post-podcast test showed significant (p<0.01) and substantive knowledge improvement (20% increased average MCQ score). Both groups showed a significant knowledge improvement suggesting that participants gained knowledge about the topic through the podcast and not only through the undergraduate curriculum. This knowledge acquisition is present despite the majority of students being engaged in other activities while listening to the podcasts.

Incorporating student podcast usage habits and preferences in creation of content

One of the major aims of our study was to help guide content creators in tailoring podcasts to the intended audience. In our specific undergraduate medical student group, two major points stood out as areas of consideration for podcast preferences. Eighty-five percent of students with a stated preference in length of podcasts stated they preferred podcasts 30 minutes long or less. FOAM producers targeting the undergraduate medical student population would benefit from recognizing this preference in order to better cater to this audience. Secondly, the majority of users stated that they were engaged in another activity while interacting with the podcast. In fact, 46% of users stated that at some point, they interacted with the podcast while driving. This information should inform producers in two ways. It should be important to recognize that the use of any sounds that are startling or similar to traffic noises (such as car horns or sirens) should be avoided to prevent confusion or safety concerns while listeners are engaging with the podcast. Moreover, there may be a utility to incorporate brief audio summaries to reinforce the material throughout the podcast, considering that listeners may not be physically taking notes for later review while engaging in the podcast. Finally, producers may want to incorporate visual or written summaries for review for reasons similar.

Limitations

This study was based out of one Canadian medical school and unfortunately had limited number of volunteer participants. Furthermore, the rate of completion following initial survey and test was quite low. The incompletion rate was high with students citing competing priorities or the length of the podcasts being too much of a time commitment. Of note, neither participation nor completion had any impact on participants’ academic standing, which could have contributed to this high attrition rate. Moreover, as this study only applied to elective podcast usage, it is difficult to translate the data to podcast use that is incorporated into a curriculum.

Furthermore, once again due to the participant selection process, a selection bias may have occurred to recruit students already interested in using podcasts as a tool for learning. In addition, these students may have been able to afford more time to listen to podcasts than students who chose not to participate in this study. While that may be the case, the significantly high drop-out rate in this study, along with the qualitative survey responses, provides useful information to help podcast producers understand the usage preferences of undergraduate medical students.

The researchers also recognize a possibility of priming effect as the baseline test and the follow-up test had the exact same questions. It is possible that students used audio cues to improve their answers to the test but not necessarily improve knowledge. Also, secondary to the nature of the downloadable format of the podcast, we were unable to prevent students from listening to the podcast while doing the test, which could possibly contaminate our knowledge acquisition results.

Unfortunately, there was not an established control group in this study to truly assess for knowledge acquisition from the podcast. It is well known that testing itself may result in more robust learning, so test-enhanced learning effects may have resulted in unintentional learning [12-13]. While we were able to demonstrate an improvement in test score pre- and post-interaction with the podcasts, the lack of a control group (i.e., students who were only asked to answer test questions) limits our ability to interpret this data.

Finally, in order to assess retention, we used a strict analysis that only included results from participants who completed them at least 48 hours after the initial baseline test. This strict analysis moderately decreased our response rate for our knowledge quizzes.

Future directions

Considering the limitations, this study serves as a starting point, identifying the usage habits and preferences of audio-only podcasts for co- or extra-curricular learning in an undergraduate medical school. There are numerous increasingly used novel education modalities that may benefit from a similar analysis. Blog posts are a rapidly growing form of FOAM that learners at various levels are using to supplement their learning. We hope to use the data and limitations from this study, in order to extend the research to include other learning resources and continue to provide education material producers with knowledge and guidance on how their audience is interacting with these resources. 

## Conclusions

Audio podcasts can be a resource for extracurricular learning in medical education. While interacting with the podcasts, the majority of students were simultaneously engaged in other activities (top three activities: driving, completing chores, and exercising). Students indicated that they preferred podcasts of 30 minutes or shorter and felt that visuals or a concluding summary would benefit their learning. We also found in this study that students who engage in such extracurricular learning successfully improved in test scores after listening to an audio podcast on a particular topic. We hope that this study will serve to guide educators in optimally creating learning resources for medical learners.

## Figures and Tables

**Figure 1:**
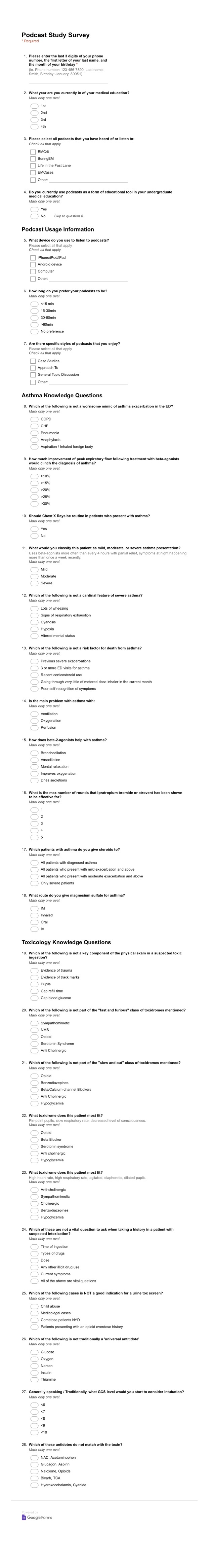
Podcast initial survey Initial survey sent to participants prior to receiving access to podcasts.

**Figure 2:**
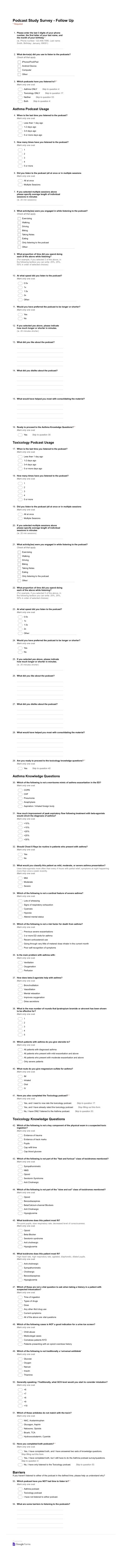
Follow-up survey Survey sent to participants 1 week after receiving access to podcasts.
